# Transcriptomic alterations in the sweet orange vasculature correlate with growth repression induced by a variant of citrus tristeza virus

**DOI:** 10.3389/fmicb.2023.1162613

**Published:** 2023-04-17

**Authors:** Vicken Aknadibossian, Jose C. Huguet-Tapia, Victor Golyaev, Mikhail M. Pooggin, Svetlana Y. Folimonova

**Affiliations:** ^1^Department of Plant Pathology, University of Florida, Gainesville, FL, United States; ^2^PHIM Plant Health Institute, University Montpellier, CIRAD, INRAE, IRD, Institute Agro, Montpellier, France

**Keywords:** RNA virus, closterovirus, citrus tristeza virus, plant virus, transcriptome, small RNA, papain-like cysteine proteases, plant-virus interactions

## Abstract

Citrus tristeza virus (CTV, family *Closteroviridae*) is an economically important pathogen of citrus. CTV resides in the phloem of the infected plants and induces a range of disease phenotypes, including stem pitting and quick decline as well as a number of other deleterious syndromes. To uncover the biological processes underlying the poorly understood damaging symptoms of CTV, we profiled the transcriptome of sweet orange (*Citrus sinensis*) phloem-rich bark tissues of non-infected, mock-inoculated trees and trees singly infected with two distinct variants of CTV, T36 or T68-1. The T36 and T68-1 variants accumulated in the infected plants at similar titers. With that, young trees infected with T68-1 were markedly repressed in growth, while the growth rate of the trees infected with T36 was comparable to the mock-inoculated trees. Only a small number of differentially expressed genes (DEGs) were identified in the nearly asymptomatic T36-infected trees, whereas almost fourfold the number of DEGs were identified with the growth-restricting T68-1 infection. DEGs were validated using quantitative reverse transcription-PCR. While T36 did not induce many noteworthy changes, T68-1 altered the expression of numerous host mRNAs encoding proteins within significant biological pathways, including immunity and stress response proteins, papain-like cysteine proteases (PLCPs), cell-wall modifying enzymes, vascular development proteins and others. The transcriptomic alterations in the T68-1-infected trees, in particular, the strong and persistent increase in the expression levels of PLCPs, appear to contribute to the observed stem growth repression. On the other hand, analysis of the viral small interfering RNAs revealed that the host RNA silencing-based response to the infection by T36 and that by T68-1 was comparable, and thus, the induction of this antiviral mechanism may not contribute to the difference in the observed symptoms. The DEGs identified in this study promote our understanding of the underlying mechanisms of the yet unexplained growth repression induced by severe CTV isolates in sweet orange trees.

## 1. Introduction

Citrus tristeza virus (CTV), a member of the family *Closteroviridae*, is considered the most economically important citrus virus, which causes significant losses to the citrus industry. The symptoms of CTV infection range from leaf cupping and epinasty, leaf vein clearing or corking, seedling yellows, and stunting to the two most damaging syndromes—quick decline and stem pitting (Dawson et al., 2015). Quick decline is an outcome of the infection of citrus varieties grafted on the sour orange rootstock by certain CTV variants. The specific virus variant-host combinations lead to rootstock-scion incompatibility, with the development of phloem necrosis below the bud union and loss of feeder roots, limiting the root capacity to supply water and nutrients, followed by tree decline and death. Stem pitting affects sweet orange, grapefruit, and lime trees irrespective of the rootstock and is a result of disrupted vascular tissue development in the stem areas invaded by the virus. Consequently, these areas appear as sunken grooves on the stem. Severe stem pitting leads to poor tree growth and loss of vigor and yield. The sour orange rootstock was largely abandoned in the citrus-growing areas with endemic decline-causing CTV variants after it was discovered to be a necessary factor for quick decline. However, CTV still causes major economic losses for citrus growers through stem pitting, stunting, yield reduction, and poor fruit quality (Moreno et al., 2008; Dawson et al., 2015).

CTV is a phloem-limited virus and possesses the largest non-segmented RNA genome (19.3 kb) among plant viruses. The flexuous filamentous CTV particle encloses a positive-sense single-stranded RNA genome that contains 12 open reading frames (ORFs) (Karasev et al., 1995; Martelli et al., 2012; Folimonova, 2020). The CTV-encoded proteins function in virus replication (ORF1a and ORF1b products), virus assembly and movement (major coat protein, minor coat protein, p65, p61, and p6), and suppression of antiviral RNA silencing (CP, p20, and p23). In addition, CTV encodes three unique proteins (p13, p18, and p33) that extend the host range of CTV and are needed for infection of selective citrus varieties (Karasev et al., 1995; Satyanarayana et al., 2000; Lu et al., 2004; Dolja et al., 2006; Tatineni et al., 2008, 2011; Agranovsky, 2016).

Presently, variants of CTV are classified into at least eight genotype groups (strains)—T3, T30, T36, T68, VT, RB, HA 16–5, and S1—on the basis of full genome sequences, which show >7.5% difference at the nucleotide level (Harper, 2013; Dawson et al., 2015; Yokomi et al., 2018). How different CTV variants cause symptoms remains largely unexplained. Most strains of CTV comprise several variants, which produce different phenotypes upon infection. Moreover, the symptoms are not only dependent on the variant of CTV but also on the citrus host. A CTV variant that is asymptomatic in one host can kill another. Furthermore, often, field CTV isolates contain populations of multiple strains (Dawson et al., 2015), making it difficult to determine the effect of each CTV variant in the mixed population on the tree symptomatology. Hence, the phenotypes of CTV infection are greatly diverse (Dawson et al., 2015).

To assess the biological pathways involved in the development of symptoms upon CTV infection, we analyzed the host responses of sweet orange (*Citrus sinensis*) seedlings infected with two different isolates of CTV exhibiting contrasting phenotypes—T36 (Karasev et al., 1995; Satyanarayana et al., 1999) and T68-1 (Folimonova et al., 2010; Harper, 2013). In sweet orange, the isolate T68-1 of the T68 strain was reported to cause moderate stem pitting, while variants of the T36 strain were known to be generally asymptomatic (Nikolaeva et al., 1998; Albiach-Marti et al., 2004). Oranges represent more than 50% of the global citrus production and over 40% of world citrus exports (FAO, 2021). Thus, a better understanding of how certain CTV variants cause deleterious symptoms in sweet orange, while others are asymptomatic, could serve as an important step toward managing CTV-induced losses in this economically valuable crop.

Each of the T36 and T68-1 isolates studied here carries a single variant of the respective strain, which eliminates any interactions resulting from potential mixed infections and allows to attribute the observed characteristics to a specific virus variant. The host response was examined by Illumina sequencing analysis of host transcriptomic changes and viral small RNA production during early stages of infection in the T36-or T68-1-inoculated sweet orange plants. Since CTV is essentially a phloem-limited virus, we chose to analyze samples extracted from the phloem-rich bark tissue of young shoots. Symptoms induced by the virus variants in the inoculated trees were monitored for 2 years. To the best of our knowledge, this is the first study that compares transcriptomic changes induced by CTV variants of the T36 and T68 strains in *C. sinensis* vascular tissue using Next Generation Sequencing and correlates those with differential symptoms produced by the two virus variants and, specifically, with growth repression induced by the variant of the T68 strain.

## 2. Materials and methods

### 2.1. Plants and virus inoculation

The Florida CTV isolates T36 (GenBank accession number: AY170468) and T68-1 (GenBank accession number: JQ965169) used in this study have been maintained in *C. macrophylla* plants under greenhouse conditions. For each treatment (Mock, T36, and T68-1), three biological replicates, in the form of 2 year-old *C. sinensis* cv. Madam Vinous seedlings, were graft-inoculated with tissue from the source plants infected with either T36 (Karasev et al., 1995; Satyanarayana et al., 1999), T68-1 (Folimonova et al., 2010; Harper, 2013), or healthy tissue. The inoculated plants were maintained under greenhouse conditions.

### 2.2. RNA extraction, library preparation, and sequencing

Phloem-rich bark tissue (100 mg) was collected from comparable young stems of new flushes when they were no longer soft and easy to peel. The tissue was ground in liquid nitrogen, and total RNA was extracted using the Direct-zol RNA MiniPrep Plus kit (Zymo Research), following the manufacturer’s instructions. The RNA was eluted in 50 μL of nuclease-free water, and the RNA profile and integrity were analyzed using Agilent Technologies Tapestation. For transcriptomic analysis, libraries were prepared using the TruSeq Stranded mRNA Library prep kit (Illumina) and sequenced on the NovaSeq 6000 S4 (Illumina) platform with 150 bp paired end reads generating a total output of more than 40 million reads per sample (Table 1). For small RNA (sRNA) analysis, libraries were prepared using the NEXTflex Small RNA Seq Kit v3 (PerkinElmer) and sequenced on the HiSeq2500 Rapid Mode 50SE (Illumina) platform with 50 bp single end reads generating a total output of 14–45 million reads per sample.

**Table 1:** Summary of assembled transcriptomes and RNA integrity number (RIN) value of sequenced RNA samples.

**Sample**	**Total reads**	**GC (%)**	**Q30 (%)**	**Overall read mapping rate (%)**	**Concordant pair alignment rate (%)**	**RIN**
*Mock_1*	49,925,454	43.76	96.04	89.0	83.7	7.8
*Mock_2*	45,126,036	44.07	95.93	89.3	84.0	7.5
*Mock_3*	43,922,146	43.69	93.96	86.9	81.4	7.8
*T36_1*	44,614,454	43.97	95.92	89.3	83.8	8.0
*T36_2*	52,346,362	43.94	95.38	88.8	82.9	8.1
*T36_3*	44,889,246	43.85	96.1	89.0	83.6	8.0
*T68-1_1*	43,985,540	44.01	93.88	88.3	82.5	7.9
*T68-1_2*	57,520,434	44.07	93.63	88.0	82.1	7.7
*T68-1_3*	43,259,470	43.62	93.93	87.9	82.2	8.1

Q30: The percentage of bases with a Phred value >30.

### 2.3. Data analysis

The mRNA-seq reads were trimmed using Trim Galore (Krueger, 2015). FastQC (Andrews, 2010) was used to analyze the reads and determined the average Phred score of 35–36 across all the samples. The reads were mapped to the reference *C. sinensis* cv. Valencia draft genome v2.0 (Xu et al., 2013) using TopHat2 (Kim et al., 2013; Table 1). Fold changes in the mapped genes were analyzed using DESeq 2 (Love et al., 2014). Differentially expressed genes (DEGs) were defined by a filtering cut-off of Padj ≤ 0.05 and log2 FC ≥ |1|.

### 2.4. Quantitative reverse transcription-PCR (RT-qPCR)

For RT-qPCR validation of the stranded mRNA-seq data, cDNA was generated from the RNA samples using the GoScript™ Reverse Transcription System (Promega) with Oligo(dT) primers following manufacturer’s instructions. Approximately 80 ng of the cDNA was used as template in 10 μL qPCR reactions (5 μL AzuraView™ GreenFast qPCR Blue Mix LR (Azure Genomics), 250 nM forward primer, 250 nM reverse primer). The sequences of primers used are provided in Supplementary Table S1. The reactions were carried out in CFX Connect™ Real-time PCR detection system (BIO-RAD) with cycling conditions of 95°C for 2 min followed by 39 cycles of 95°C for 5 s and 60°C for 28 s. Fold changes were calculated with gene specific PCR efficiency correction and two reference gene normalization as described (Hellemans et al., 2007).

To determine the virus titer of the T36 and T68-1 variants in the inoculated plants, one-step RT-qPCR was performed with primer/probe pairs specific to either T36 or T68-1 variants as described in Bergua et al. (2016). RNA concentration was determined using a NanoDrop Ultraviolet–Visible (UV–Vis) Spectrophotometer (Thermo Fisher Scientific, Waltham, MA, USA). Standard curves were prepared with 10-fold dilutions ranging from 10^9^ to 10^5^ copies of a target transcript according to the procedure described in Bergua et al. (2016).

### 2.5. Small RNA analysis

The Illumina generated sRNA reads were trimmed to remove NEXTflex sequencing adapters using cutadapt^1^ and mapped to the reference sequences of the CTV T36 (GenBank accession number: AY170468) and T68-1 (GenBank accession number: JQ965169) variants and *C. sinensis* cv. Valencia nuclear (GenBank assembly accession: GCA_022201045.1), chloroplast (GenBank accession: DQ864733.1), and mitochondrial (GenBank accession: MG736621.1) genomes with Burrows-Wheeler Aligner (BWA).^2^ The BWA alignment files (.bam) were used as inputs to generate CTV consensus sequences for each dataset with MISIS-2 (Seguin et al., 2016). Both viral and plant sRNA reads mapped without mismatches to the CTV consensus sequences and *C. sinensis* nuclear, chloroplast, or mitochondrial genomes, respectively, were sorted according to their size class (15–34 nt), polarity, and 5′-nucleotide identity and counted (Supplementary Dataset S1). The read counts in each library were normalized in reads per million (RPM) of total (15–34 nts) reads. The distribution of viral sRNA reads along the CTV genomes and their 5′-nucleotide identity profiles were analyzed and visualized with MISIS-2 (Seguin et al., 2016; Supplementary Dataset S1 and Supplementary Figure S1). Single nucleotide resolution maps of the sRNA reads mapped to the CTV genomes with zero mismatches are provided in Supplementary Dataset S2.

## 3. Results and discussion

### 3.1. Infection by the two CTV variants resulted in differential manifestation of symptoms

The goal of this study was to analyze differences in the host responses induced upon inoculation of sweet orange seedlings by an asymptomatic T36 variant or a more damaging T68-1 variant and correlate those to the symptoms observed upon tree infection. Accordingly, 2 year-old *C. sinensis* cv. Madam Vinous plants were grafted-inoculated with the tissue from either healthy trees (mock) or source plants infected with either T36 or T68-1 variants of CTV. Each treatment had three biological replicates.

**Figure 1:**
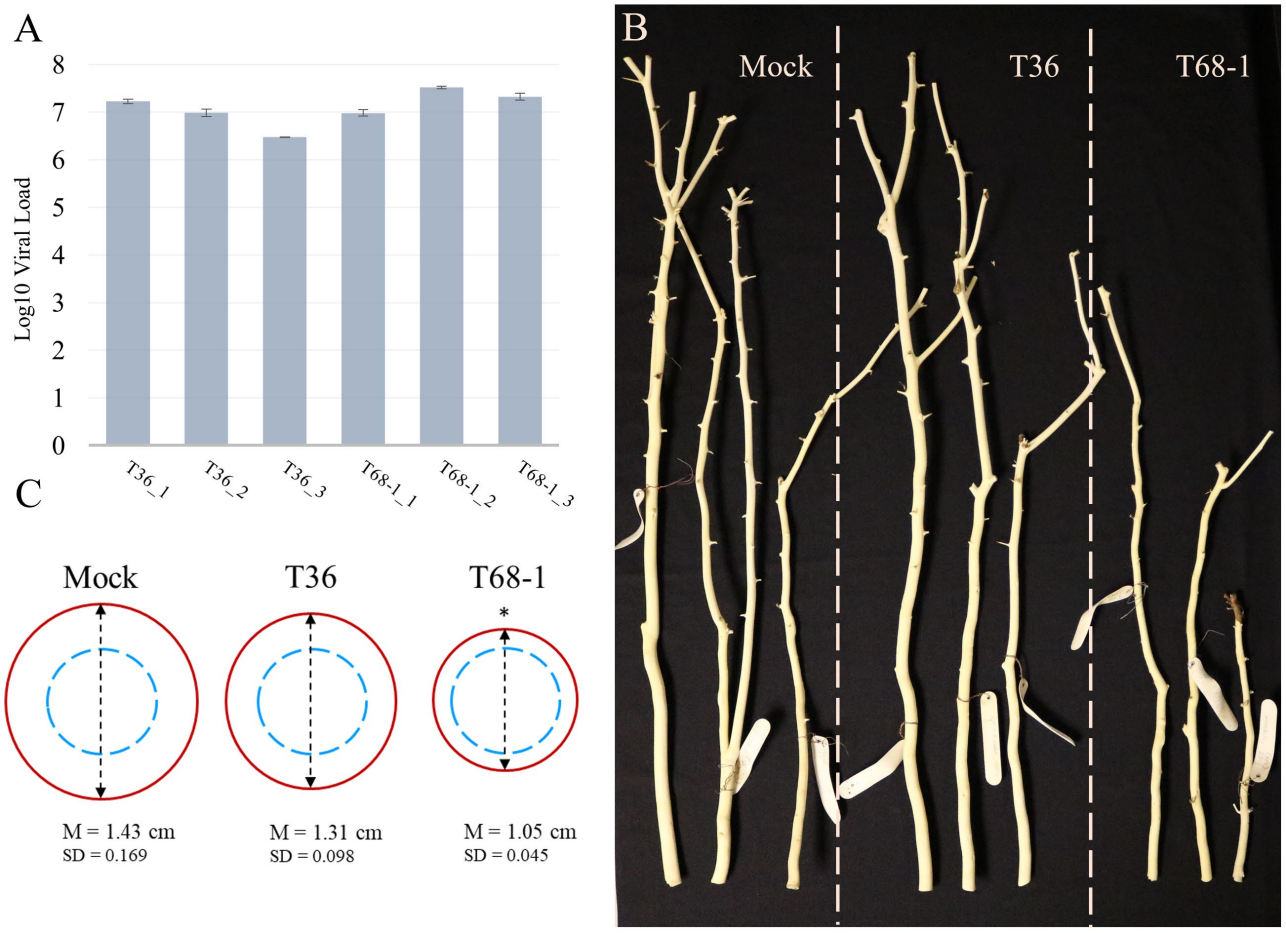
Virus titer and symptoms produced in mock-, T36-, and T68-1-inoculated *Citrus sinensis* trees. **(A)** Absolute quantification of the virus titer in T36- and T68-1-infected samples determined by RT-qPCR using T36-or T68-1-specific primers and probes (viral load = number of genomic RNA copies per ng of total RNA). Error bars represent the standard deviation between the three technical replicates of each sample. No statistically significant differences as determined by Student’s *t*-test were found between the T36 and T68-1 samples (*p* > 0.2). **(B)** Comparison of the size and diameter of the main stem of mock-, T36-, or T68-1-inoculated trees at two years post-inoculation. Bark was peeled to assess stem pitting. **(C)** Average diameter of wood at the base of a tree after removal of the bark. Dashed blue circles represent the comparable starting diameter of all samples at the time of graft inoculation. Red circles represent the measured diameter two years post-inoculation. *The mean stem diameter of T68-1-infected trees is significantly different than that in Mock, value of *p* (0.0148) < 0.05. M, mean value, SD, standard deviation.

At 4 months post-inoculation, the establishment of systemic infections was evaluated *via* absolute quantification of the virus titer using RT-qPCR and total RNA extracted from the bark tissue of young stems of the T36-and T68-1-inoculated plants. The results confirmed that the replicates of the trees inoculated with T36 and T68-1 became infected at comparable virus titers (Figure 1A). No statistically significant differences as determined by Student’s *t*-test were found between the T36 and T68-1 samples (*p* > 0.2).

At the time of sample collection (4 months post-inoculation), the trees inoculated by T36 or T68-1 as well as mock treatments did not exhibit any obvious symptoms. No stem pitting was observed on mock-and T36-inoculated sweet orange plants. The T68-1-inoculated trees did not show any detectable stem pits either. As the infections progressed, no stem pitting was detected in any of the trees, even at two years post-inoculation. The ability of the T68-1 variant used in our inoculations to induce stem pitting in sweet orange has been reported (Nikolaeva et al., 1998; Albiach-Marti et al., 2004). With that, the age of the trees, the growth conditions, and the time required to develop these symptoms were not specified in these studies. Such factors, however, could play an important role. For example, while quick decline of sweet orange grafted on the sour orange rootstock is a devastating outcome of decline-causing isolates of CTV in the field trees, the syndrome cannot be replicated in small trees grown under the greenhouse conditions (Dawson et al., 2015). Similarly, the reason behind the lack of apparent stem pitting in our T68-1-infected trees might be a combination of young seedling age, insufficient incubation time, greenhouse growth conditions, or other factors.

Examination of the trees in the different treatments over the course of 2 years revealed that the T68-1-inoculated trees had a slower growth rate when compared to mock-or T36-inoculated trees (Figure 1B). The growth repression was obvious both in the general height of the trees as well as the diameter of the stem (Figure 1C; the stem diameter of the trees was measured at the base of each tree after peeling off the bark, which was performed to assess for stem pitting, at 2 years post-inoculation). Stunting is a common CTV phenotype that can result from many host-virus variant combinations and even be an outcome of two distinct syndromes such as stem pitting and seedling yellows. Severe stem pitting can cause stunting, yet stunting and stem pitting are not always closely correlated (Garnsey et al., 1987). For example, in a study examining CTV stem pitting and stunting in Madam Vinous sweet orange, two of the infected trees received the same rating of moderately stunted. However, while one of them was found to have “many deep” stem pits, the other only developed “few shallow” ones (Williams et al., 2002). The severity of the stunting observed in the T68-1-infected trees can be regarded as an almost complete cessation of the radial stem growth, with the stem thickness after 2 years being only marginally larger than that at the time of grafting. In contrast, mock-and T36-infected trees displayed a considerable amount of growth (Figure 1C).

### 3.2. Overview of the RNA-seq data, transcriptomic analysis, and RT-qPCR validation

Total RNA was extracted from the bark tissue samples of young shoots at 4 months post-inoculation. This time period allows sufficient time for the virus to establish systemic infection but is still considered an early infection stage. Symptoms such as stem-pitting and stunting are the result of virus interference with the normal differentiation of the vascular tissue, which is only apparent after a considerable time of further growth. Hence, the aim of this work was to discover early infection pre-symptomatic transcriptomic changes that could be the causative changes of later developed symptoms. When symptoms have already appeared, the transcriptomic changes originating from altered host anatomy/physiology could mask the causative changes.

The RNA samples were analyzed by Tapestation to determine RNA integrity values (RIN). RIN values are a measure of the level of RNA degradation with values ranging from 1 (totally degraded) to 10 (intact) (Schroeder et al., 2006). All samples had RIN values between 7.5 and 8.1, indicating that RNA degradation was minimal (Table 1). A total number of 43–57 million reads were obtained per sample, suggesting good coverage of the citrus transcriptome. A quality control score (Q30) (Sequencing Quality Scores, n.d.) greater than 93.63% and GC content of 43.62–44.07% confirmed the quality of the reads. These reads were mapped to the *C. sinensis* reference genome (Xu et al., 2013) with a concordant pair alignment rate of 81.4–84% (Table 1).

**Figure 2:**
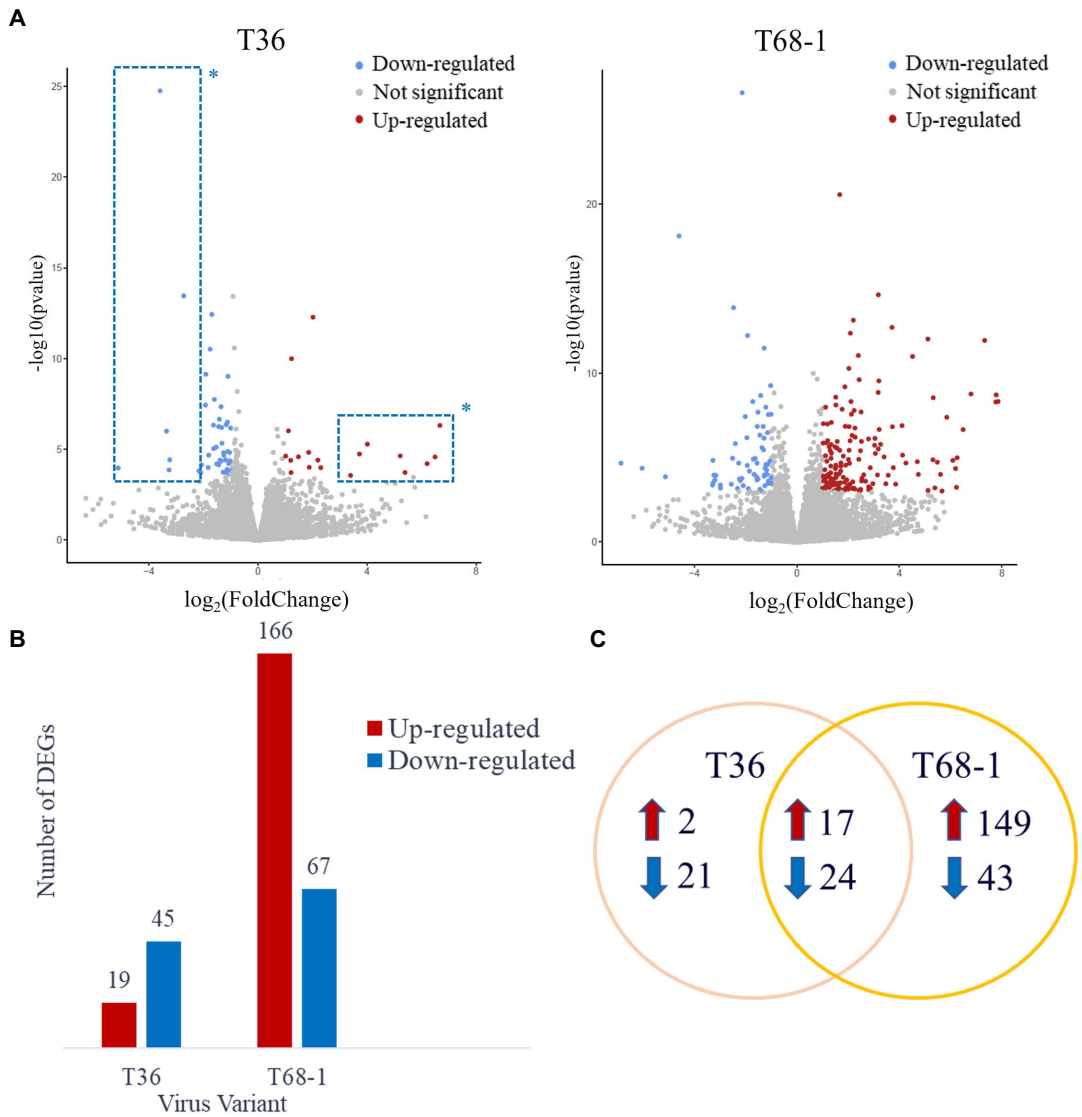
Overview of the DEGs identified in the T36- and T68-1 infected *Citrus sinensis* trees. **(A)** Volcano plots of DEGs identified in the T36- and T68-1-infected plants. Colored dots represent significant DEGs with padj < 0.05 and Log2FC > |1|. Blue dots represent down-regulated DEGs, and red dots represent up-regulated DEGs. *Dots inside the dashed boxes represent some of the DEGs identified in the T36-infected trees that were also identified in the T68-1-infected trees. **(B)** Bar graph summarizing the number of up-regulated and down-regulated DEGs in the T36-and T68-1-infected trees. **(C)** Venn Diagram illustrating the number of unique and shared DEGs in the T36-and T68-1-infected trees.

Host differentially expressed genes (DEGs) were identified by comparing the expression profiles in trees inoculated with T36 and T68-1 to those in mock-inoculated trees (Figure 2A). The T36 infection resulted in a total of 64 DEGs (19 up-regulated, 45 down-regulated), while the T68-1 infection resulted in 233 DEGs (166 up-regulated, 67 down-regulated), almost fourfold more than that by T36 (Figure 2B). Notably, a stronger impact of the T68-1 infection on the host transcriptome correlated with the retardation of plant growth, which resulted in a marked stunting of trees over the course of infection. The association of the number of DEGs and the severity of symptoms has been reported for viral infections of *Nicotiana benthamiana* and *Solanum lycopersicum*. Generally, an asymptomatic infection that did not greatly disturb host anatomy and physiology appeared not to alter the host transcriptome significantly. Conversely, more severe symptoms correlated with pronounced transcriptomic changes (Pesti et al., 2019). This observation also agrees with comparative transcriptomic studies with other CTV variants in other hosts (Gandía et al., 2007; Khalilzadeh et al., 2022). For instance, a much higher number of DEGs was reported in *C. macrophylla* infected with a CTV p33 deletion mutant (CTVΔp33) causing severe stem pitting, compared to a mild stem pitting-inducing wild type CTV. On the flip side, almost no DEGs were observed with the asymptomatic p13 deletion mutant (CTVΔp13) (Khalilzadeh et al., 2022). A similar result was found in *Citrus aurantifolia* where no DEGs were identified upon infection with the mild T385 isolate, while hundreds of DEGs were found with the severe T305 isolate (Gandía et al., 2007). Yet, another study reported a similar number of DEGs in sweet orange leaves infected with either a mild T30 variant or a severe VT variant (Fu et al., 2016). Such differences in how different citrus hosts are impacted by CTV variants suggest that transcriptomic findings in one host with one CTV variant may not be assumed in others.

Importantly, most of the DEGs identified with the T36 infection were also identified with the T68-1 infection (41 DEGs). Many of the DEGs that were identified upon infection with both T36 and T68-1 have been also found to be impacted by other plant viruses (discussed below), and are generally not implicated in pathways critical to host development. On the contrary, the biological processes involving DEGs identified only in the T68-1-infected trees encompassed vital pathways such as plant development, immunity, and stress response. Unique DEGs identified in the T68-1 infection (192 DEGs) represent more than an eightfold increase, compared to those of the T36 infection (23 DEGs) (Figure 2C). In addition, the eight most up-regulated and seven most down-regulated DEGs identified upon the T36 infection were also identified with the T68-1 infection (Figure 2A, dashed boxes). Hence, the T36-specific DEGs were few and not very strongly altered. Overall, these data clearly demonstrate a much stronger alteration of the host transcriptome by the T68-1 infection, compared to that of T36. A full list of T36-and T68-1-specific DEGs is presented in Supplementary Table S2.

Some of the proteins encoded by the DEGs did not have any significant homology with proteins in the annotation databases and were classified as uncharacterized proteins (Supplementary Table S2). The high fold change in the expression of some of these DEGs suggests that they are heavily involved in the virus-host interactions. Unfortunately, their role cannot be defined any further before they are characterized.

To validate the RNA-Seq data, changes in the expression of 14 *C. sinensis* genes identified as DEGs were determined by RT-qPCR (Supplementary Table S1). UBQ10 and ACTB were chosen as two citrus internal reference genes (Yan et al., 2012). The expression levels of these 14 genes in the T36-and T68-1-infected samples relative to mock-inoculated samples determined by RT-qPCR were similar to those observed in the mRNA-Seq data (Supplementary Table S1). A high Pearson correlation coefficient (*r* = 0.90) between the results of the two methods validated the accuracy of the RNA-Seq data.

### 3.3. Both variants of CTV altered the expression of a narrow set of common genes

**Figure 3:**
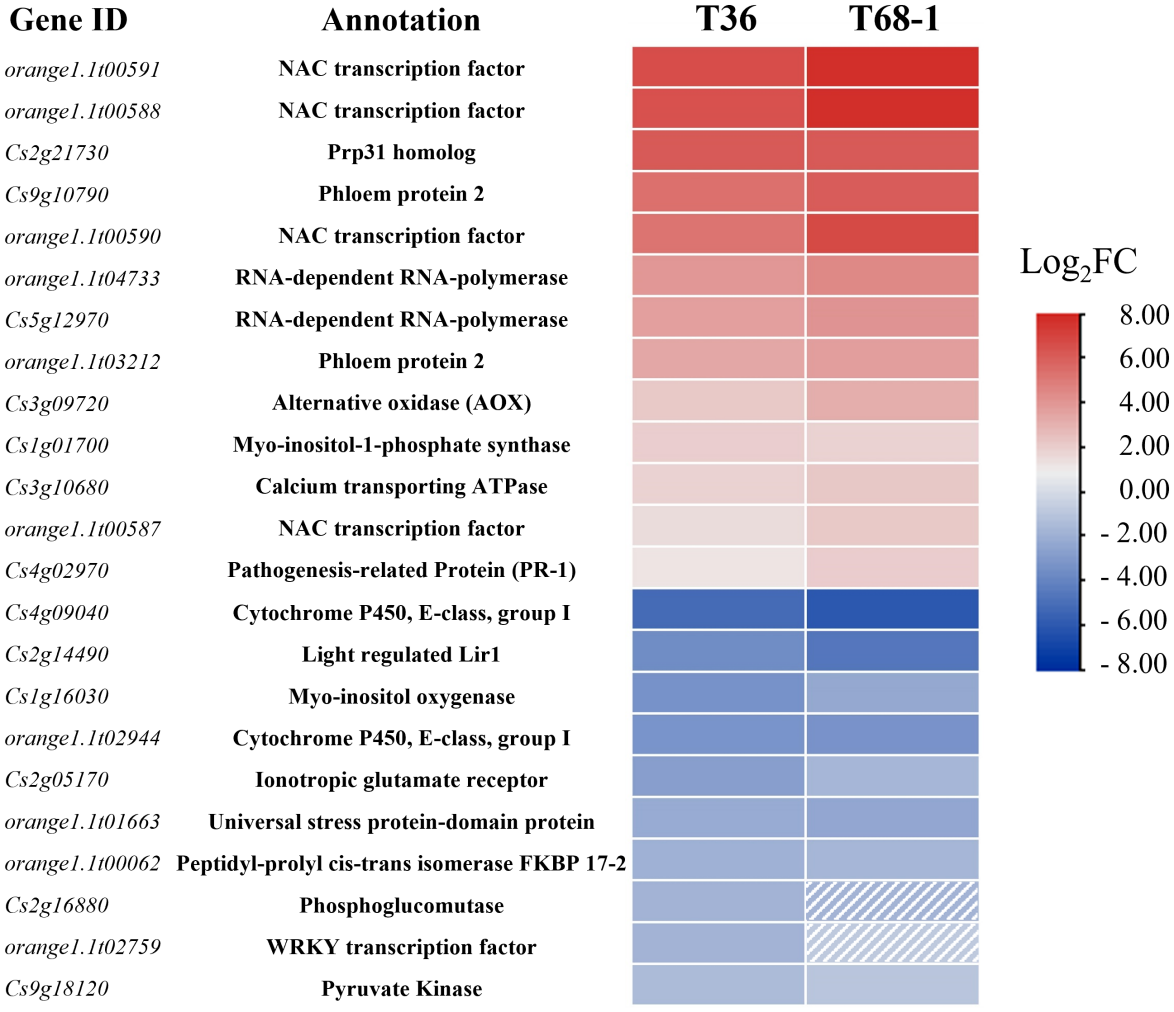
Heatmap of the most up-regulated and down-regulated DEGs in the T36-infected samples and their respective expression levels in the T68-1-infected samples. Dashed boxes are used to indicate genes that are not differentially expressed in the T68-1-infected samples (padj > 0.05 or Log2FC < |1|).

As T36 and T68-1 are both variants of CTV, they were expected to impact a common set of DEGs that can be regarded as a general host response to CTV infection. These DEGs included 17 up-regulated and 24 down-regulated genes. Some of the highly up-regulated and down-regulated DEGs are presented in Figure 3.

Among the most up-regulated DEGs found with both variants were four NAC (NAM, ATAF, and CUC) transcription factors (TFs). Two of them (orange1.1t00591 and orange1.1t00588) represent DEGs strongly up-regulated by both CTV variants (Figure 3). The T68-1 infection also induced two additional NAC TFs (Cs1g09640 and Cs6g14240). NAC TFs were found to regulate plant resistance against viruses such as tomato yellow leaf curl virus (TYLCV) and rice dwarf virus (Bian et al., 2020). Furthermore, NACs have been reported to be hijacked by viruses. The interaction of different plant virus proteins [wheat dwarf virus RepA, tobacco mosaic virus (TMV) helicase, tomato leaf curl virus replication enhance protein, and turnip crinkle virus CP] with NAC TFs were found to greatly impact virus infection, in some cases, negatively and, in others, positively (Yuan et al., 2019). Given such commonality of plant virus-NAC TF interactions and the high induction of multiple NAC TFs in both T36-and T68-1-infected trees, it is very likely that NAC TFs play a role in the CTV infection cycle.

Other significant DEGs identified with both CTV variants could aid our understanding of conserved CTV-citrus interaction mechanisms, whether they can be considered pro-viral or anti-viral (Figure 3). A few noteworthy examples are listed below.

#### 3.3.1. Pro-viral DEGs

Both T36 and T68-1 induced the levels of the transcripts coding for the phloem protein 2 (PP2) and an alternative oxidase (AOX). The PP2 family comprises phloem-limited proteins that have been shown to bind to mRNAs (Ham et al., 2009), viroids (Gómez and Pallás, 2001; Owens et al., 2001), and cucurbit aphid borne yellows virus (CABYV) virions (Bencharki et al., 2010). In *Arabidopsis thaliana*, CABYV virion binding to PP2s was shown to protect the virions from degradation and improve transmission rates (Bencharki et al., 2010). PP2s can also modify the size-exclusion limit of the plasmodesmata and move cell-to-cell (Balachandran et al., 1997). Some have suggested that PP2s are involved in the long-range trafficking of RNA molecules such as viroids (Pallas and Gómez, 2013; Ma and Wang, 2022). The above research and the induction of PP2s in the T36-and T68-1-infected trees suggest that PP2s could also have a pro-viral role in CTV infection. The T68-1 infection up-regulated two additional PP2s (Cs9g10760 and Cs9g10910). The AOX proteins are known to suppress the accumulation of reactive oxygen species (ROS) during virus infection (Mayers et al., 2005; Zhang et al., 2012). In fact, in our earlier study, AOX-1a was found to be greatly induced during the infection by a T36 variant of CTV to mitigate (ROS) response in *N. benthamiana* leaves (Kang et al., 2019). Therefore, the induction of the transcripts of two PP2s and AOX identified in this study appears to have pro-viral implications (Figure 3).

#### 3.3.2. Anti-viral DEGs

It is not surprising that the genes of RNA-dependent RNA polymerases (RDRs) were induced by both CTV variants as they were shown to be up-regulated during infection with a number of other viruses, including TMV, cucumber mosaic virus (CMV), and sugarcane mosaic virus as part of the plant RNA silencing-based antiviral response (Khan et al., 1986; Xie et al., 2001; Yu et al., 2003; He et al., 2010; Qin et al., 2017; Basu et al., 2018; Leibman et al., 2018). On the other hand, the mRNA levels for FKBP 17–2 and a pyruvate kinase decreased during infection by both T36 and T68-1 (Figure 3). Earlier, it was found that the CTV protein p23 interacts with FKBP 17–2 and changes its localization from chloroplasts to plasmodesmata in *N. benthamiana*. Furthermore, the CTV CP also interacts with FKBP 17–2, and p23 alters the localization of the CP/FKBP17-2 complex. Knocking down the FKBP 17–2 mRNA resulted in significantly lower accumulation of CTV (Yang et al., 2021). Pyruvate Kinase was found to be recruited by tomato bushy stunt virus into the viral replication complex to supply an ample amount of ATP utilized by DEAD-box helicases to boost viral RNA replication (Chuang et al., 2017). Thus, the down-regulation of these proteins upon the T36 and T68-1 infection could represent manifestation of the host antiviral defense.

### 3.4. Infection by the T68-1 variant up-regulated expression of genes coding for papain-like cysteine proteases (PLCPs)

**Figure 4:**
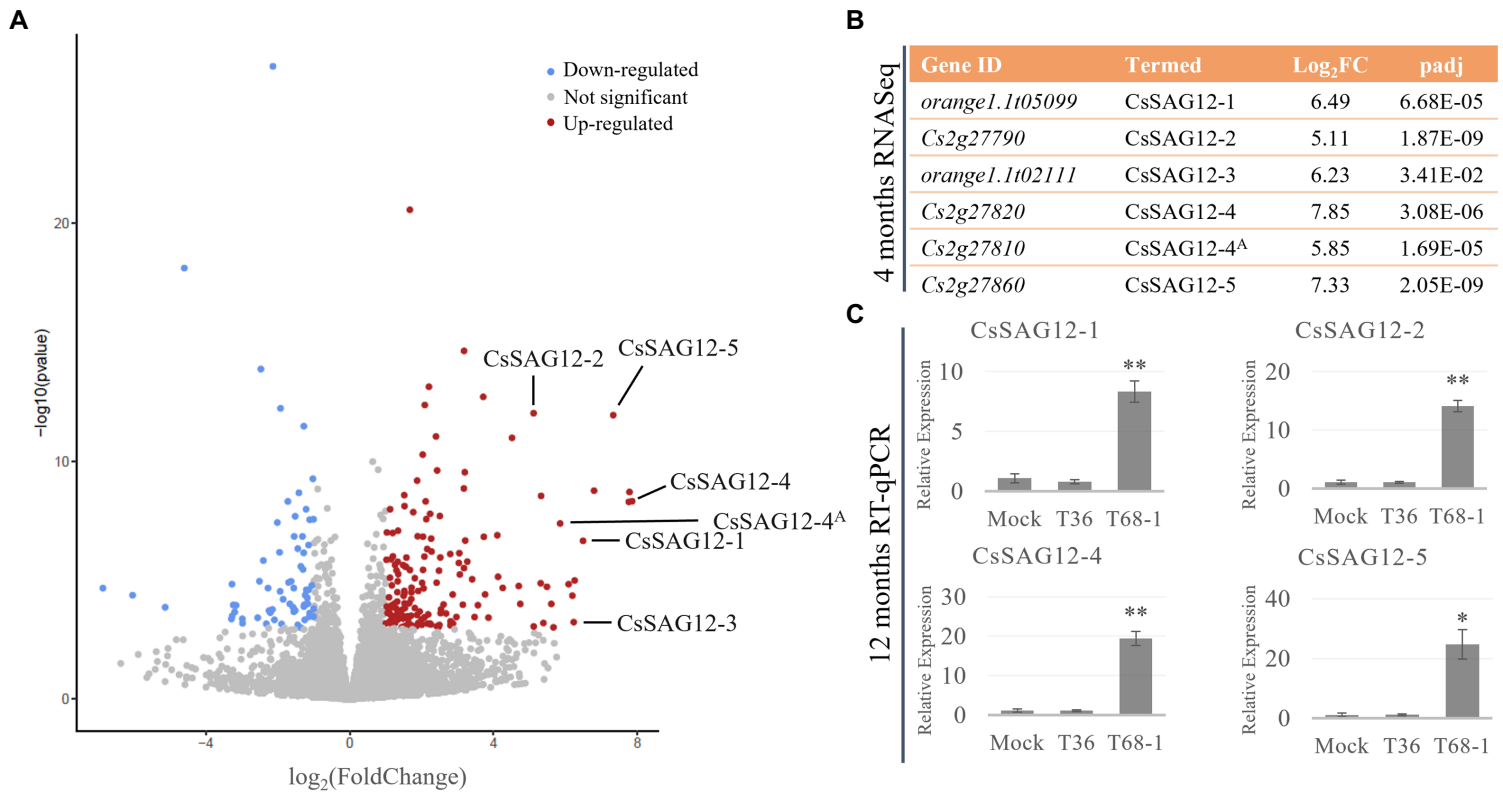
CsSAG12 PLCPs are strongly up-regulated throughout the course of T68-1 infection in *Citrus sinensis*. **(A)** Volcano plot of DEGs identified in T68-1-infected trees, illustrating significantly higher up-regulation of CsSAG12 PLCPs relative to other genes in the host transcriptome. **(B)** PLCPs identified as the CsSAG12 family members and their expression level as determined by RNA-Seq at 4 months post-inoculation. **(C)** Expression levels of CsSAG12 family members as determined by RT-qPCR at 12 months post-inoculation. “A” indicates the Cs2g27820 and Cs2g27810 paralogs representing CsSAG12-4. “*” indicates significant difference (*p* < 0.05), “**” indicates extremely significant difference (*p* < 0.01) as determined by Student’s *t*-test.

In the T68-1-infected trees, genes encoding six papain-like cysteine proteases (PLCPs) were some of the most strongly induced genes (Figure 4A). Upon the analysis of their amino acid sequences on the NCBI BLASTP platform (Altschul et al., 1990) using the non-redundant UniProtKB/SwissProt database, the two highest scored hits for all six PLCPs were found to be the *A. thaliana* senescence-specific cysteine protease (AtSAG12) and xylem cysteine peptidase 1 (AtXCP1). Both SAG12 and XCP1 belong to the C1A family of vacuolar PLCPs. Even though the closest ortholog was AtSAG12 for all PLCPs, the percentage identity was only between 48.4 and 53.7%. It should be noted that Cs2g27820 and Cs2g27810 have 99.4% amino acid identity, coding for almost identical proteins. However, Cs2g27810 lacks a canonical PLCP N-terminal signal peptide. Thus, Cs2g27810 and Cs2g27820 could be considered paralogs.

Interestingly, the same PLCPs were reported to be involved in the *C. sinensis* response to another phloem-limited pathogen. Sec-delivered effector 1 (SDE1), an effector of the Huanglongbing-associated bacterium ‘*Candidatus* Liberibacter asiaticus’ (CLas), was found to directly interact with the protease domain of multiple *C. sinensis* PLCPs and inhibit their activity. Phylogenetic analysis of these PLCPs with members of the *A. thaliana* PLCP families placed some of them in the SAG12 family (Clark et al., 2018). Four of the SAG12 proteins that were reported to bind SDE1 are the same PLCPs induced by T68-1 in this study. Accordingly, the PLCPs identified here are termed as described in the above study as CsSAG12-1 (orange1.1t05099), CsSAG12-2 (Cs2g27790), CsSAG12-3 (orange1.1t02111), CsSAG12-4 (Cs2g27820; Cs2g27810). An additional SAG12-like PLCP up-regulated by T68-1, which was not described in the above study, is designated as CsSAG12-5 (orange1.1t05099) (Figure 4B).

Furthermore, RT-qPCR assays with the primers targeting the CsSAG12 transcripts (Supplementary Table S1) were performed with RNA collected from the T36-, T68-1-, and mock-inoculated trees at 1 year post-inoculation. The results confirmed strong induction of CsSAG12 transcripts in the vascular tissue not only at 4 months but also at 1 year post-inoculation in the T68-1-infected trees (Figure 4C). This demonstrates that such up-regulation of the PLCP genes was not a temporary effect but rather has continued throughout the course of infection.

PLCPs have roles in many biological processes in plants such as development, immunity, stress responses, germination, and senescence (Liu et al., 2018). The SAG12 family is best known for its high expression during leaf senescence and is often used as a senescence marker. In rice, *sag12* mutant lines exhibited delayed senescence. AtSAG12 was reported to act in Rubisco degradation during leaf senescence and nitrogen remobilization during low nitrogen conditions to sustain seed production (James et al., 2018). AtSAG12 was also found to be expressed in the stele of the roots where it facilitated proteolysis to free nitrogen (James et al., 2019). Although members of the SAG12 family were first identified as senescence-related proteases, subsequent reports have shown their involvement in plant immunity. Members of the SAG12 family, Rcr3 and Pip1, were found to participate in plant defense in tomato. Both proteases were inhibited by the *Cladosporium fulvum* effector Avr2. Rcr3 acts as a co-receptor for *Cf*-2 dependent hypersensitive response triggered by Avr2. *Rcr3* mutants also show more susceptibility to *Phytophthora infestans* in the absence of *Cf*-2. Pip1, on the other hand, is a broad-range immune protease, and its depletion leads to significantly increased susceptibility to fungal, oomycete, and bacterial pathogens (Ilyas et al., 2015). Rcr3 and Pip1 were also inhibited by the *Pseudomonas syringae* effector Cip1 (Shindo et al., 2016). With that, the full spectrum of biological processes they are involved in is still unknown. Particularly, the effect of the SAG12 protease abundance or activity on stem or vascular tissue development has not been studied. In addition, while SAG12 family members are primarily implicated in senescence and nitrogen remobilization in *A. thaliana* and plant immunity in tomato, they might have different roles in citrus.

As mentioned above, the CLas effector SDE1 can bind the protease domain of many *C. sinensis* PLCPs (Clark et al., 2018). CLas infection was found to induce CsSAG12s at both the transcriptomic (Kim et al., 2009; Martinelli et al., 2012) and proteomic levels (Clark et al., 2018). However, the activity of CsSAG12s was not altered by CLas infection. This suggests that, even though they could be involved in CLas defense, their activity might be suppressed by the pathogen (Clark et al., 2018). While CLas infection is typically associated with a range of leaf and fruit symptoms and eventual tree decline, prolonged infection also causes stunting of infected trees (Serrano et al., 2010). It is intriguing that both CLas and T68-1 induce CsSAG12s, and both cause stunting of the affected trees.

The CsSAG12s only have about 50% amino acid sequence identity with the AtSAG12 protein. Although this homology is considerable, it is not sufficient to decisively classify them as the SAG12 family proteins. The protein with the second highest BLASTP score according to the UniProtKB/SwissProt database (also with ~50% amino acid identity to the PLCPs up-regulated by T68-1) is AtXCP1. XCP1 is localized to the vacuoles of differentiating tracheid elements (TEs). Ectopic expression of XCP1 in *A. thaliana* resulted in decreased plant size and chlorophyll levels, which correlated with increased XCP1 peptidase activity (Funk et al., 2002). TE autolysis is the programmed cell death of maturing xylem cells to transform into empty TEs. This autolysis was proposed to be caused by cysteine proteases and hydrolases loaded into the vacuole and released during vacuole implosion (Kuriyama and Fukuda, 2002). XCP1 was also found to have a major role in micro-autolysis (Avci et al., 2008).

PLCPs can play important roles in plant-virus interactions. The TYLCV V2 protein was found to bind and inhibit the protease activity of CYP1 PLCP (Bar-Ziv et al., 2015). The turnip mosaic virus (TuMV) 6 k1 protein, on the other hand, was rapidly degraded by host PLCPs when ectopically expressed in the absence of TuMV. When co-expressed with TuMV, 6 k1 was stable and significantly reduced protease activity in the host, leading to increased TuMV accumulation (Bera et al., 2022). T68-1 strongly and persistently induced five CsSAG12 PLCPs. It is conceivable that they act in senescence and/or broad or effector-triggered immunity, similar to SAG12 proteases. It is also conceivable that they act in TE autolysis and maturation, similar to XCP1 proteases. Given the repression of radial stem growth observed in the T68-1-infected trees, it would be tempting to propose the latter as solely responsible for the observed stunting. However, the cause of the repression of the radial stem growth and stunting is likely the outcome of not just CsSAG12s, but also other groups of DEGs described below.

### 3.5. T68-1 had a prominent impact on genes coding for cell-wall modifying enzymes (CWMEs) and proteins involved in vascular development

**Figure 5:**
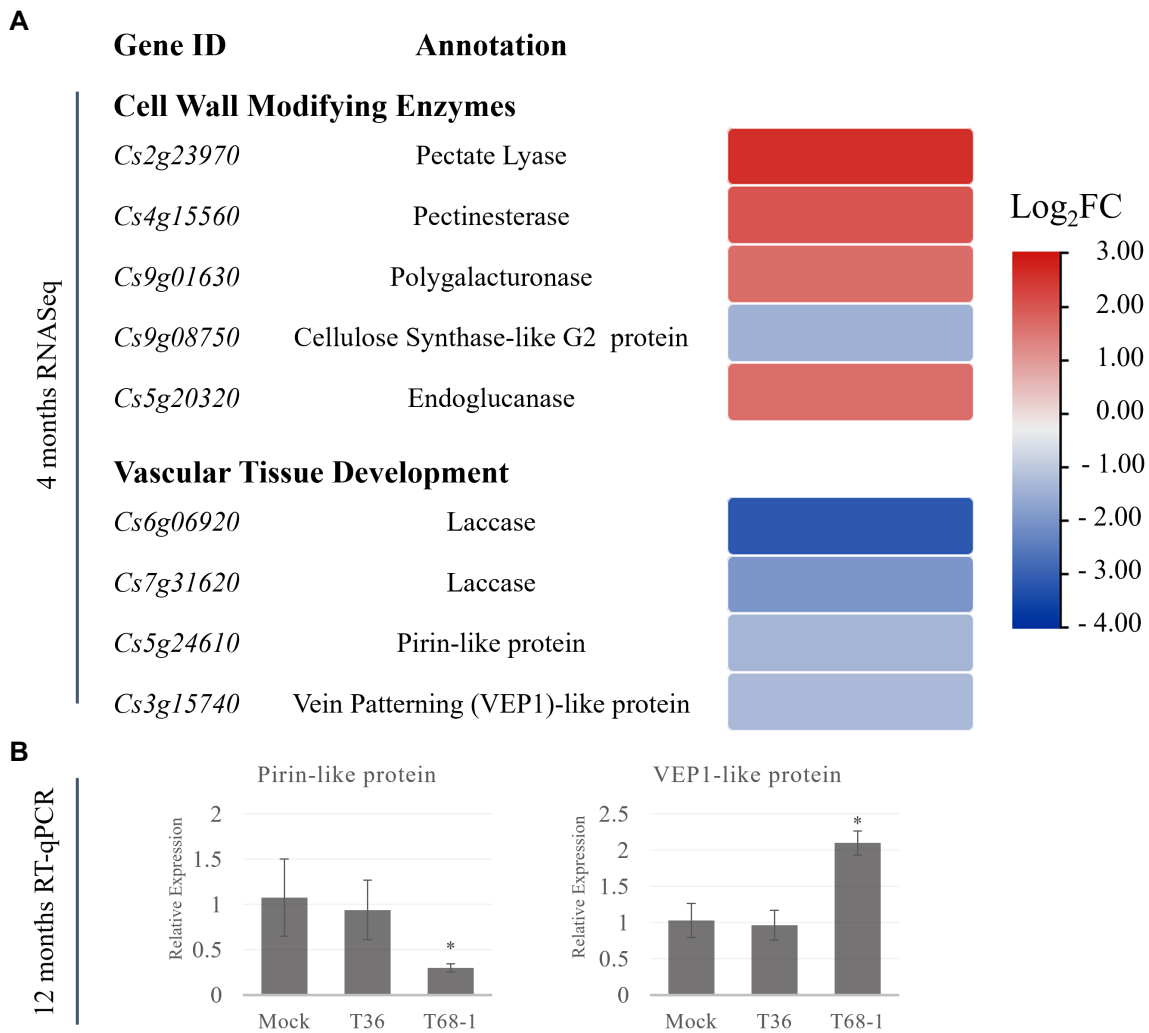
Expression of DEGs representing cell-wall modifying enzymes and DEGs involved in the vascular differentiation in T68-1-infected samples. **(A)** Heatmap of DEGS at 4 months post-inoculation. **(B)** Relative expression of selected genes at 12 months post-inoculation assayed by RT-qPCR. “*” indicates significant difference (*p* ≤ 0.05) as determined by Student’s *t*-test.

In the T68-1-infected trees, several CWME DEGs were identified. These DEGs are involved in the secondary cell wall and vascular tissue development, which can be linked to the plant growth repression observed with the T68-1 infection. These DEGs include the up-regulated pectin hydrolyzing enzymes with roles in plant development and secondary wall formation such as a pectate lyase, a pectinesterase, a polygalacturonase, and an endoglucanase (Figure 5A; Glass et al., 2015; Yang et al., 2018). Interestingly, overexpression of poplar pectate lyase 18 resulted in the reduction of the secondary cell wall thickness and irregular xylem cells (Uluisik and Seymour, 2020).

Alteration of CWME expression patterns have been reported for many plant viruses. For example, CWMEs were found to be induced in cucumber green mottle mosaic virus-infected watermelon flesh (Li et al., 2017) and mungbean yellow mosaic India virus-infected soybean (Yadav and Chattopadhyay, 2014). Infection by beet necrotic yellow vein virus (BNYVV) produced severe symptoms, including reduced size, deformation, and necrosis of the vascular tissue. Interestingly, a comparative transcriptomic study found that the number of DEGs involved in the cell wall organization or biogenesis was more than twofold higher with BNYVV, compared to the milder beet soil-borne mosaic virus (Fernando Gil et al., 2020). This correlation between CWMEs induction and virus-induced host growth repression parallels our observations in the T68-1-infected trees.

In addition, a number of DEGs with demonstrated roles in vascular tissue development were down-regulated by the T68-1 infection. These include two laccases, a pirin-like protein, and a 3-oxo-delta (4,5)-steroid 5-beta-reductase known as a vein patterning (VEP1)-like protein (Figure 5A). Laccases are necessary for lignin polymerization and normal vascular development in *A. thaliana* (Zhao et al., 2013). Down-regulation of a laccase in poplar led to severe deformation of xylem fiber cell walls (Ranocha et al., 2002). Laccases also play a role in disease resistance in cotton: a laccase was strongly induced by a *Verticillium* infection, which resulted in the increase of cell wall lignification as a part of the host defense (Zhang et al., 2019). *A. thaliana* PIRIN2, which shares 71% amino acid identity with the pirin-like protein identified in *C. sinensis* in this study, suppressed syringyl (S)-type lignin accumulation in the secondary cell walls of the xylem vessels and increased susceptibility to *Ralstonia solanacearum* (Zhang et al., 2014, 2020). *A. thaliana* VEP1 was demonstrated to be heavily involved in the vascular tissue development and maintenance. Mutant *vep1* plants had markedly reduced stem thickness with the number of the procambial, xylem, and phloem cells being considerably reduced during early stem development. In the mature stem and root, the secondary growth from the cambial cells was limited (Jun et al., 2002). The *C. sinensis* VEP1-like protein shares 71% amino acid identity with AtVEP1.

In contrast to down-regulation of two laccases by T68-1 in our study, CLas was reported to induce three laccases, while variants of the T30 and VT strains of CTV, similarly to T36 in this study, did not alter the expression of laccases in *C. sinensis* leaves (Fu et al., 2016). Conversely, a group of DEGs involved in “xylem specification” was found to be associated with the invasion of both the full-length T36 and CTVΔp33 in *C. macrophylla* (Khalilzadeh et al., 2022). DEGs in this group included pectate lyases, pectinesterases, a polygalacturonase, and four laccases. As shown above, genes from these protein families are also identified as DEGs in the T68-1 infection in *C. sinensis*. However, the specific genes identified in the two studies are not the same. The same *C. sinensis* genome was used to map reads in both studies. Therefore, a possibility of variations originating from different annotations can be excluded. All CWMEs identified by Khalilzadeh et al. (2022) were up-regulated, similarly to the T68-1-induced CWMEs. The T68-1 infection in *C. sinensis* down-regulated two laccases. Khalilzadeh et al. (2022) found the full-length T36 and CTVΔp33 infections up-regulated three laccases in *C. macrophylla* and CTVΔp33 down-regulated an additional laccase. None of these DEGs were identified with the asymptomatic CTVΔp13 (Khalilzadeh et al., 2022) or T36 in our study.

The genes encoding pirin-like and VEP1-like proteins have not been identified as DEGs in any previous work exploring the effect of CTV infection on citrus transcriptomes. Given the novelty of this finding, the status of the expression of these genes was investigated at the 1 year timepoint. Similar to what was found for the earlier infection timepoint (e.g., 4 months post inoculation), both genes were expressed at comparable levels in the mock-inoculated and the T36-infected trees at 12 months post inoculation, while they remained differentially expressed in the T68-1 infected trees (Figure 5B). The pirin-like protein gene remained down-regulated at the 1 year timepoint, yet the expression of the VEP1-like protein gene was altered from being down-regulated at 4 months to being up-regulated at 1 year (Figure 5B). This data suggested that the suppression of the pirin-like protein is a sustained effect throughout the course of the T68-1 infection. On the other hand, the VEP1-like protein appears to be a more dynamic host factor that can be up-or down-regulated at different stages of the T68-1 infection. Overall, the alteration of the expression of CWMEs and the genes involved in the vascular differentiation could disturb normal vascular tissue development. Upon reviewing previous work examining the responses of various citrus hosts to different variants of CTV, we found that these groups of DEGs were particularly altered in the CTV-citrus host combinations where disturbances in the vascular tissue development such as stem pitting or repression of the radial stem growth were observed. Furthermore, the differential expression of the pirin-like and VEP1-like proteins observed at both 4 and 12 months post virus inoculation, appears to be T68-1-specific as they have not been reported in any other CTV-related transcriptomic studies. The CWMEs and the vascular differentiation-associated DEGs are likely a contributing factor to the repression of growth observed in the T68-1-infected trees.

### 3.6. Several DEGs involved in disease resistance, signaling, and stress response were associated with the T68-1 infection

Plant defense relies on the effector-triggered immunity (ETI) and pathogen-associated molecular pattern-triggered immunity (PTI) (Jones and Dangl, 2006). ETI is triggered by sensor nucleotide-binding domain leucine-rich repeat (LRR)-containing (NLR) proteins. Many plant NLRs have been reported to recognize viral proteins and function in defense against viruses (Ngou et al., 2022). TIR-NBS-LRR receptors have been reported to confer N gene-mediated resistance to TMV (Peart et al., 2005) and Ry_sto_ resistance to potato virus Y (Grech-Baran et al., 2020). Receptor-like proteins (RLPs) and receptor-like kinases (RLKs) act as pattern recognition receptors and constitute an integral part of PTI (Böhm et al., 2014; Yu et al., 2017). RLPs and RLKs have been shown to function in virus resistance as well. For instance, the BAK1 RLK mutant showed decreased resistance to several RNA viruses in *A. thaliana* (Kørner et al., 2013); OsRLP1 modulated rice resistance against rice black-streaked dwarf virus (Zhang et al., 2021); and CaRLPs were involved in the pepper immune response against TMV and pepper mottle virus (Kang et al., 2022). Furthermore, induction of R-related genes has been previously reported in plant-virus interactions, particularly, when the infection was accompanied by severe symptoms or the hypersensitive response was triggered (Li et al., 2018; Kalapos et al., 2021). Interestingly, in some cases, viral proteins recognized by plant NLRs triggering antiviral ETI serve as pathogenicity determinants and suppressors of RNA silencing (Zvereva and Pooggin, 2012).

Given the importance of these proteins in host-virus interactions, DEGs identified with both CTV variants were analyzed for resistance (R) gene-related domains and motifs using DRAGO 3 (Disease Resistance Analysis and Gene Orthology) pipeline of plant resistance gene database (PRGDB) (Calle García et al., 2022). Out of the unique 149 DEGs up-regulated in the T68-1 infection, 34 possessed R-gene related domains (Table 2). The majority were transmembrane receptors with 12 RLPs and 10 RLKs, and seven were classical R proteins with TIR-NBS-LRR-TM domains (TNL). T68-1 also down-regulated one TNL. The most up-regulated DEGs were RLPs and RLKs, while TNLs were more modestly up-regulated (Table 2). In addition to classical R-related domains identified by DRAGO3, T68-1 also up-regulated an enhanced disease susceptibility 1 (EDS1)-like protein. While not an NLR, EDS1 is required by all tested TNLs in dicots for resistance and programmed cell death (Lapin et al., 2020). This is in agreement with the up-regulation of seven TIR-NLRs by T68-1. As for T36, the only up-regulated R-related gene was the weakly up-regulated TM-kinase, which was also up-regulated by T68-1 (Cs4g01450). However, three R-related DEGs were slightly down-regulated during the T36 infection. These include a mitogen-activated protein kinase, a cysteine-rich RLK, and a TM-LRR (Supplementary Table S2).

**Table 2:** Prediction of R gene domains/motifs present in the T68-1-specific DEGs using DRAGO 3 tool of plant resistance gene database.

**Gene ID**	**Class**	**Domain type**	**Log**_**2**_ **FC**	**padj**
*Cs1g22350*	TNL	NBS-LRR-TM-TIR	1.74	0.0432
*orange1.1t04913*	TNL	NBS-LRR-TM-TIR	1.52	0.0095
*orange1.1t01840*	TNL	NBS-LRR-TM-TIR	1.50	0.005
*orange1.1t01844*	TNL	NBS-LRR-TM-TIR	1.30	0.0007
*Cs5g20360*	TNL	NBS-LRR-TM-TIR	1.30	0.0134
*orange1.1t04750*	TNL	NBS-LRR-TM-TIR	1.19	3.97E-05
*orange1.1t04573*	TNL	NBS-LRR-TM-TIR	1.17	0.0119
*Cs1g14770*	RLP	LRR-TM	5.66	0.0478
*Cs3g06270*	RLP	LRR-TM	3.56	0.0105
*Cs9g12040*	RLP	LRR-TM	2.80	0.0418
*orange1.1t04439*	RLP	LRR-TM	2.79	0.0002
*Cs4g10640*	RLP	LRR-TM	2.49	0.0406
*Cs6g12330*	RLP	LRR-TM	2.48	0.0007
*Cs9g12240*	RLP	LRR-TM	2.27	0.0002
*Cs3g06220*	RLP	LRR-TM	2.26	0.04
*Cs5g20370*	RLP	LRR-TM	2.08	0.0002
*orange1.1t03142*	RLP	LRR-TM	1.66	0.0375
*Cs6g12310*	RLP	LRR-TM	1.48	0.0267
*Cs1g22410*	RLP	LRR-TM	1.20	0.0272
*Cs2g29920*	RLK	TM-Kinase-LRR	5.38	0.0362
*Cs1g14280*	RLK	TM-Kinase-LRR	4.70	0.0024
*Cs9g03360*	RLK	TM-Kinase-LRR	2.84	0.0376
*Cs7g14240*	RLK	TM-Kinase-LRR	2.53	0.0183
*Cs7g24650*	RLK	TM-Kinase-LRR	2.15	0.0001
*Cs1g09190*	RLK	TM-Kinase-LRR	2.13	1.20E-05
*Cs2g31130*	RLK	TM-Kinase-LRR	1.87	0.021
*orange1.1t03079*	RLK	TM-Kinase-LRR	1.72	0.0027
*orange1.1t04419*	RLK	TM-Kinase-LRR	1.71	0.0221
*Cs2g12140*	RLK	TM-Kinase-LRR	1.33	0.0038
*orange1.1t04443*	NL	NBS-LRR-TM	3.05	0.0002
*Cs5g06160*	N	NBS-TM	1.12	0.0324
*orange1.1t03411*	KIN	TM-Kinase	4.25	0.0027
*Cs9g15620*	KIN	TM-Kinase	2.18	0.0201
*Cs2g23540*	KIN	TM-Kinase	2.00	0.0283
*Cs2g22370*	KIN	TM-Kinase	1.33	0.0004
*Cs4g01450*	KIN	TM-Kinase	1.02	0.0003

**Figure 6:**
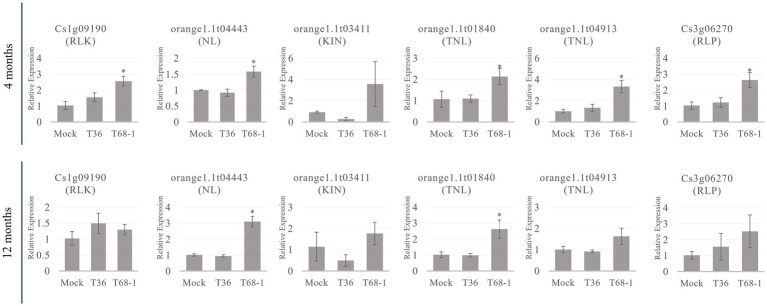
RT-qPCR relative expression at the 4 and 12 months timepoints of R-related genes from different classes of R-related genes (RLK, NL, KIN, TNL, RLP) identified as DEGs in T68-1 infected samples. “*” indicates significant difference (*p* ≤ 0.05) as determined by Student’s *t*-test.

Since R-related genes make up a large portion of the DEGs in the T68-1 infected trees, the expression levels of members belonging to each class (listed in Table 2) of R-related genes (a RLK, a NL, a KIN, two TNLs, and a RLP) were determined by RT-qPCR at the 4 and 12 months timepoints (Figure 6). All six genes were up-regulated in the T68-1 infected trees at 4 months post inoculation, confirming the RNA-Seq analysis. The same trend was observed at the 1 year timepoint for five of the six genes in the T68-1-infected plants. The expression levels of these genes in the T36-infected trees remained comparable to those of mock-inoculated trees at both timepoints. This finding further strengthens the possibility of R genes playing a key role in the *C. sinensis* interactions with the T68-1 CTV variant, which elicited a more pronounced phenotypic response, compared to that of the asymptomatic T36 variant. Interestingly, it was reported that a mild CTV variant of the T30 strain induced 14 R proteins, while a severe one from the VT strain only induced one R protein in *C. sinensis.* On the other hand, the severe VT-like variant up-regulated eight RLPs, while the mild T30-like induced only one (Fu et al., 2016). Furthermore, a full-length T36 variant was found to repress one and induce two R proteins and strongly induce six RLKs in *C. macrophylla*. The same group also found that CTVΔp33, which caused severe stem pitting, elevated five and repressed 13 R proteins, yet no genes coding RLKs or RLPs were affected (Khalilzadeh et al., 2022). These opposing observations, along with our results, led us to believe that there is no common citrus R protein, RLP, or RLK that mediate the response to all CTV variants. Additionally, such responses seem to be specific to certain combinations of CTV variants and citrus hosts.

Several stress response genes, including chaperone-encoding genes, were affected primarily by T68-1 (Supplementary Table S2). While T36 down-regulated a DnaJ 11-like protein, T68-1 up-regulated DnaJ 3-and DnaJ 19-like proteins and down-regulated a DnaJ 6-like protein. Many DnaJ-like host proteins have been reported to interact with plant virus proteins to influence virus infection (Hofius et al., 2007; Shimizu et al., 2009; Cho et al., 2012; Verchot, 2012; Zong et al., 2020; Liu et al., 2022). Three HSP20, one HSP70, and one HSP90 genes were moderately up-regulated by T68-1. This phenomenon has been reported for many plant virus infections upon which induction of heat shock proteins led to an increase of viral genomic RNA, proteins, or virion accumulation and facilitated infection (ul Haq et al., 2019).

Despite the substantial number of DEGs implicated in stress and immune response altered by T68-1, the titers of T36 and T68-1 were similar in the host, and there was no effective resistance to T68-1. On the contrary, the fitness cost of inducing so many defense-related genes may have contributed to the growth retardation of the T68-1-infected plants.

### 3.7. CTV variants impacted expression of diverse miscellaneous genes with potential pro-or anti-viral roles

In addition to the groups of DEGs discussed above, few other interesting DEGs with previously reported roles in host-virus interactions were observed in the T68-1-and T36-infected plants. These do not fit the above categories and are reported separately below.

#### 3.7.1. T68-1-specific DEGs with possible pro-viral effects

Based on reports from other virus-host pathosystems, the up-regulation of 60S ribosomal protein L19 (RPL19), high mobility group-box (HMGB) protein, and GL2-expression modulator (GEM) and the down-regulation of two carboxylesterases detected upon the T68-1 infection can be interpreted as pro-viral responses. The accumulation of both TMV and TuMV was shown to be dependent on several ribosomal proteins, including RPL19 (Yang et al., 2009). Bamboo mosaic virus (BaMV) can interact with the *N. benthamiana* high mobility group nucleoprotein (NbHMG1/2a) and shuttle it into the cytoplasm, which subsequently enhances the BaMV systemic movement (Alazem et al., 2020). GEMs co-localize with F-actin and play a role in the cytoskeleton remodeling through modification of the actin cytoskeleton and microtubule network. The cytoskeleton plays an important role in plant virus trafficking to the plasmodesmata, and the disruption of actin filaments impairs the infection of viruses such as TMV and TuMV (Reagan and Burch-Smith, 2020). Human T-cell leukemia virus type-1 (HTLV-1) Tax protein was shown to promote GEM expression and, as a result, improve cell migration and formation of the cell-to-cell conjugates, which led to enhanced cell-to-cell viral transmission of HTLV-1 in cell cultures (Chevalier et al., 2014). In tobacco, a carboxylesterase was shown to interact with TMV CP and act as a potent antiviral agent, inhibiting TMV infection when transiently expressed (Guo and Wong, 2020).

#### 3.7.2. T68-1-specific DEGs with possible anti-viral effects

Conversely, the down-regulation of a tetraspanin 3 and lipid transfer protein (LTP) can be regarded as anti-viral responses. Tetraspanins have been shown to be heavily involved in animal virus infection, with roles in virus entry/endocytosis, trafficking, and budding of enveloped viruses (Florin and Lang, 2018). It was recently shown that tetraspanin 3 proteins also play an important role in enhancing plant virus cell-to-cell movement and infection in *A. thaliana* (Zhu et al., 2022). LTPs are responsible for non-vesicular trafficking of lipids in cells. Both animal and plant viruses have been reported to hijack LTPs to promote infection. TMV was reported to up-regulate LTP expression in both *A. thaliana* and *N. benthamiana* (Collum and Culver, 2017). BaMV infection down-regulates a *N. benthamiana* LTP, and LTP knockdown significantly reduces BaMV accumulation (Chiu et al., 2020). Viruses with a positive-sense RNA genome were also shown to hijack the cholesterol transport system in cells, directing cholesterol to VRCs to improve replication (Wong et al., 2019).

#### 3.7.3. T36-specific DEGs

The T36 infection down-regulated a putative phosphoglucomutase (PGM). PGM was not required for the successful infection of three viruses (cauliflower mosaic virus, CMV, and turnip vein clearing virus), but *pgm1* mutants displayed less severe symptoms (Handford and Carr, 2007). It is interesting that T36 also did not induce severe symptoms and, at the same time, down-regulated PGM. The T36 variant also down-regulated a coatomer protein (COP) subunit-like protein gene. COPs are proteins that coat the vesicles trafficking between the endoplasmic reticulum and Golgi apparatus. COPI and COPII have been shown to be involved in the replication of positive-strand RNA viruses such as tombusviruses and potyviruses (Agaoua et al., 2021).

These miscellaneous DEGs observed in either T36-or T68-1-infected plants reveal many interesting parallels between CTV and other viruses, which merits further investigation that might uncover universal targets and key players in virus-host interactions.

### 3.8. RNA silencing may not contribute to the difference in the symptoms produced by the two variants of CTV

**Figure 7:**
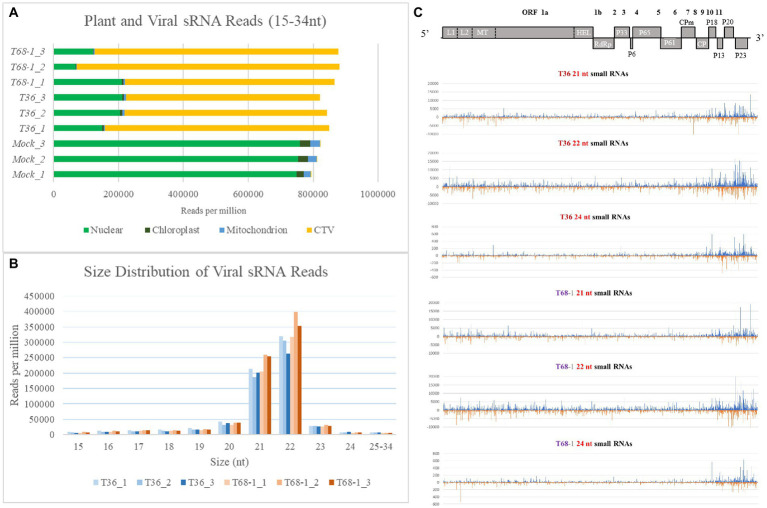
Overview of the sRNA analysis of the mock-, T36-, and T68-1 inoculated *Citrus sinensis* trees. **(A)** Number of sRNA reads per million mapped to the *Citrus sinensis* genomes (nuclear, chloroplast, or mitochondrion) and to the genomes of the CTV variants (T36 or T68-1) in mock-, T36-, and T68-1-inoculated samples. **(B)** Size distribution of viral sRNA reads in T36-and T68-1-inoculated samples. **(C)** Viral sRNA reads (21, 22, and 24 nt) in trees infected with either T36 or T68-1 mapped to their positions in their respective genomes. The above diagram represents a map of CTV ORF positions across its genome. L1 and L2, papain-like leader proteases; MT, methyltransferase; HEL, helicase; RdRp, RNA-dependent RNA polymerase; CPm, minor coat protein; CP, coat protein.

RNA silencing functions as a key antiviral immune response for virus suppression in plants. In order to determine if variations in the RNA silencing response in *C. sinensis* contributed to the observed differences in the symptoms produced by the two CTV variants, we performed sRNA sequencing and analysis using the same RNA extracts that were used to analyze the transcriptomic alterations. As discussed above, there was a clear difference in the symptoms induced by the T36 and T68-1 variants as well as in the *C. sinensis* transcriptomic responses to those viruses. Remarkably, manifestation of the host RNA silencing response was quite comparable for the two variants. When compared to mock-inoculated trees, it was clear that a robust RNA silencing response was triggered in both the T36-and T68-1-inoculated plants as represented by the overwhelming majority of sRNA reads of the viral origin (Figure 7A). The profiles of both viral and host sRNA reads in the T36-and T68-1-infected samples were similar. No substantial differences were observed in the number, size, polarity, or 5′-nucleotide identity of sRNAs derived from the *C. sinensis* genome (Supplementary Figure S1 and Supplementary Dataset S1). Furthermore, for both virus variants, the most abundant viral sRNAs of both sense and antisense polarities belonged to the 21 and 22 nt classes (Figure 7B; Supplementary Figure S1; Supplementary Dataset S1). These are the expected sizes of small interfering RNAs (siRNAs) produced by the Dicer-like (DCL) enzymes DCL4 and DCL2, which are primarily responsible for the host RNA silencing-based antiviral response (Pooggin, 2018; Ramesh et al., 2021). The siRNAs were produced from all areas throughout the entire genomes of T36 and T68-1, with a large proportion of those corresponding to the 3′-terminal regions (Figure 7C and Supplementary Dataset S1). This was in agreement with earlier reports assessing RNA silencing in citrus hosts infected with various CTV isolates (Ruiz-Ruiz et al., 2011; Folimonova et al., 2014). No substantial differences were observed in the 5′-nucleotide identity profiles of siRNAs derived from T36 and T68-1 (Supplementary Dataset S1), suggesting that Argonaute proteins sorting viral siRNAs mostly based on size and 5′-nucleotide identity (Pooggin, 2018) may not be differentially affected by the two CTV variants. Overall, no significant differences were found in the citrus RNA silencing-based responses against T36 and T68-1, suggesting that activation of this host antiviral defense mechanism may not have contributed to the observed differences in the symptoms induced by the two variants.

## 4. Conclusion

In this work, we demonstrated strong stunting and cessation of radial stem growth in *C. sinensis* seedlings infected with the T68-1 variant of CTV. Comparative transcriptomic analysis of these trees along with the trees infected with an asymptomatic T36 variant of CTV and healthy trees revealed T68-1-specific DEGs involved in vascular differentiation and plant immunity. Almost fourfold as many DEGs were identified in T68-1 infection, compared to that of T36. The strongest T68-1-specific transcriptomic changes were the CsSAG12 PLCPs. This also marks this study as the first report of the induction of PLCPs and repression of genes involved in the vascular tissue development such as the genes encoding the pirin-like and VEP1-like proteins during CTV infection. Small RNA analysis revealed that at the early infection time-point, the host RNA silencing response resulting in degradation of viral RNAs is comparable for the T36 and T68-1 variants, and thus, may not be a contributing factor to the difference in the symptoms observed at the later time-points. The cause of the radial stem growth inhibition and tree stunting is likely the cumulative outcome of the fitness cost of the up-regulated stress and immunity genes and the action of CWMEs, proteins involved in vascular development, and CsSAG12s, in particular. The strong induction of the CsSAG12s genes, that persisted throughout the course of infection with T68-1, suggests they may have had a prominent impact on the growth repression observed in the infected *C. sinensis* trees.

## Data availability statement

The datasets presented in this study can be found in online repositories. The names of the repository/repositories and accession number(s) can be found in the article/Supplementary material.

## Author contributions

SF conceived the study. VA and SF designed the research. VA, JH-T, MP, VG, and SF performed the research and analyzed the data. VA and SF wrote the manuscript with contributions from all the authors. All authors contributed to the article and approved the submitted version.

## Funding

This research was supported by the National Science Foundation (NSF) under Grant Number MCB-1615723 and in part by the United States Department of Agriculture (USDA) National Institute of Food and Agriculture, Hatch Project FLA-PLP-006024. Any opinions, findings, and conclusions or recommendations expressed in this material are those of the author(s) and do not necessarily reflect the views of the NSF and the USDA. This work of MP’s team was supported by the French National Research Agency (ANR) project ROME ANR-18-CE20-0017-01.

## Conflict of interest

The authors declare that the research was conducted in the absence of any commercial or financial relationships that could be construed as a potential conflict of interest.

## Publisher’s note

All claims expressed in this article are solely those of the authors and do not necessarily represent those of their affiliated organizations, or those of the publisher, the editors and the reviewers. Any product that may be evaluated in this article, or claim that may be made by its manufacturer, is not guaranteed or endorsed by the publisher.

## Footnotes

^1^ https://cutadapt.readthedocs.io/en/stable/

^2^ https://bio-bwa.sourceforge.net/
