# Supplementary material for: Transcriptomic alterations in the sweet orange vasculature correlate with growth repression induced by a variant of citrus tristeza virus
Source: Front Microbiol. 2023 Apr 17;14:1162613. doi: 10.3389/fmicb.2023.1162613 (PMC10150063; doi:10.3389/fmicb.2023.1162613)
Supplement: Supplementary file 1 [file Presentation_1.PPTX]

## Slide 1
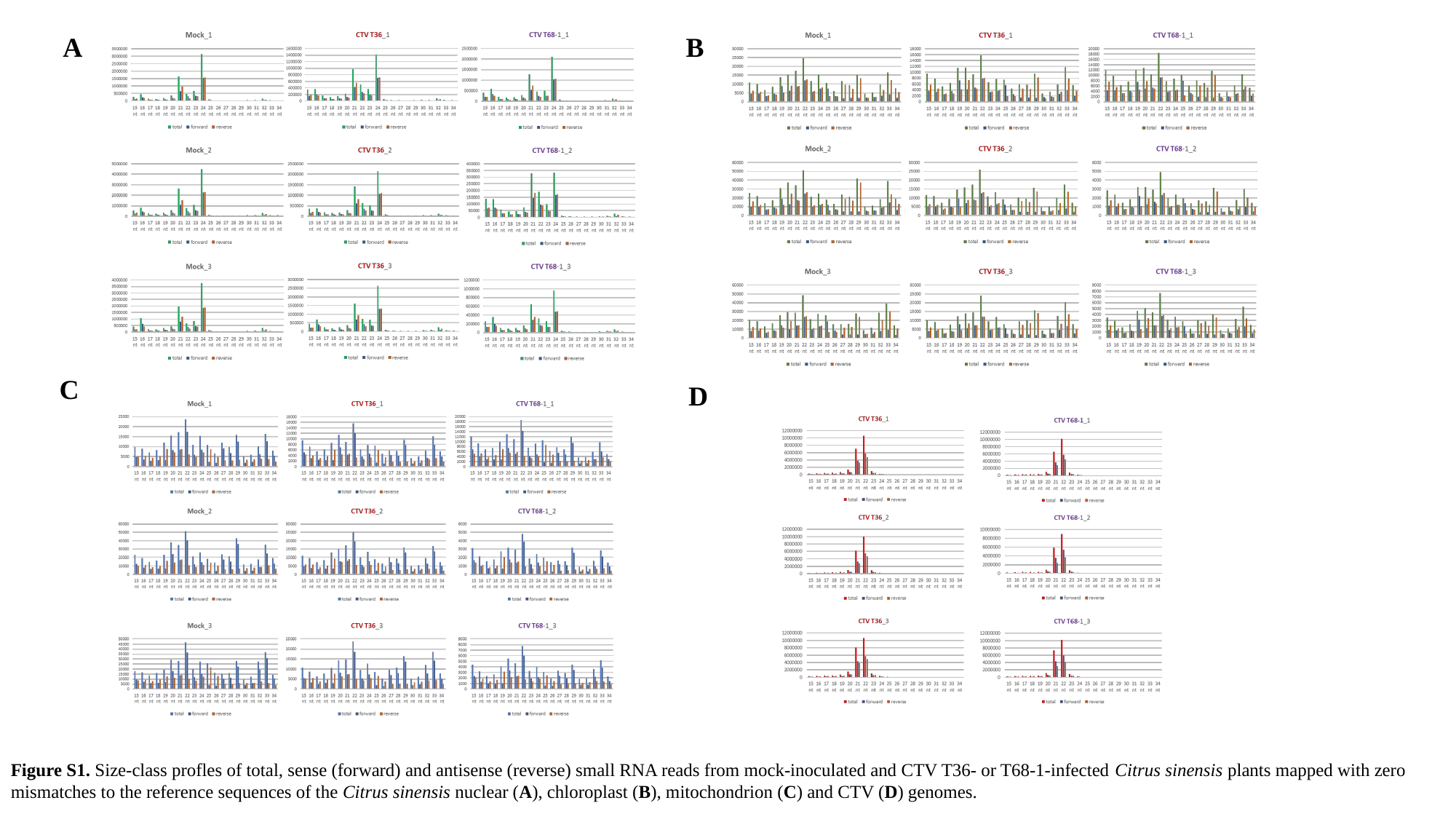

A
B
C
D
Figure S1. Size-class profles of total, sense (forward) and antisense (reverse) small RNA reads from mock-inoculated and CTV T36- or T68-1-infected Citrus sinensis plants mapped with zero mismatches to the reference sequences of the Citrus sinensis nuclear (A), chloroplast (B), mitochondrion (C) and CTV (D) genomes.
